# Unsuccessful TB treatment outcomes with a focus on HIV co-infected cases: a cross-sectional retrospective record review in a high-burdened province of South Africa

**DOI:** 10.1186/s12913-017-2406-x

**Published:** 2017-07-10

**Authors:** M. C Engelbrecht, N. G Kigozi, P. Chikobvu, S. Botha, H. C. J. van Rensburg

**Affiliations:** 1 Centre for Health Systems Research & Development, University of the Free State, P. O. Box 339, Bloemfontein, 9300 South Africa; 2 Free State Department of Health, P.O. Box 277, Bloemfontein, 9300 South Africa; 3 Department of Community Health, University of the Free State, PO Box 339, Bloemfortein, 9300 South Africa; 4 JPS Africa, Postnet Suite 132, Private Bag X14, Brooklyn, 0011 South Africa

**Keywords:** Tuberculosis (TB), TB-HIV co-infection, Unsuccessful treatment outcomes, Electronic TB register, Retrospective record review, South Africa

## Abstract

**Background:**

South Africa did not meet the MDG targets to reduce TB prevalence and mortality by 50% by 2015, and the TB cure rate remains below the WHO target of 85%. TB incidence in the country is largely fuelled by the HIV epidemic, and co-infected patients are more likely to have unsuccessful TB treatment outcomes. This paper analyses the demographic and clinical characteristics of new TB patients with unsuccessful treatment outcomes, as well as factors associated with unsuccessful treatment outcomes for HIV co-infected patients.

**Methods:**

A cross-sectional retrospective record review of routinely collected data for new TB cases registered in the Free State provincial electronic TB database between 2009 and 2012. The outcome variable, unsuccessful treatment, was defined as cases ≥15 years that ‘died’, ‘failed’ or ‘defaulted’ as the recorded treatment outcome. The data were subjected to descriptive and logistic regression analyses.

**Results:**

From 2009 to 2012 there were 66,940 new TB cases among persons ≥15 years (with a recorded TB treatment outcome), of these 61% were co-infected with HIV. Unsuccessful TB treatment outcomes were recorded for 24.5% of co-infected cases and 15.3% of HIV-negative cases. In 2009, co-infected cases were 2.35 times more at risk for an unsuccessful TB treatment outcome (OR: 2.35; CI: 2.06-2.69); this figure decreased to 1.8 times by 2012 (OR: 1.80; CI: 1.63-1.99). Among the co-infected cases, main risk factors for unsuccessful treatment outcomes were: ≥ 65 years (AOR: 1.71; CI: 1.25-2.35); receiving treatment in healthcare facilities in District D (AOR: 1.15; CI 1.05-1.28); and taking CPT (and not ART) (AOR: 1.28; CI: 1.05-1.57). Females (AOR: 0.93; CI: 0.88-0.99) and cases with a CD4 count >350 (AOR: 0.40; CI: 0.36-0.44) were less likely to have an unsuccessful treatment outcome.

**Conclusions:**

The importance of TB-HIV/AIDS treatment integration is evident as co-infected patients on both ART and CPT, and those who have a higher CD4 count are less likely to have an unsuccessful TB treatment outcome. Furthermore, co-infected patients who require more programmatic attention are older people and males.

**Electronic supplementary material:**

The online version of this article (doi:10.1186/s12913-017-2406-x) contains supplementary material, which is available to authorized users.

## Background

Tuberculosis (TB) is a major global health problem, infecting millions of people annually and ranks alongside the human immunodeficiency virus (HIV) as a leading cause of death worldwide [1]. TB and HIV are inextricably linked in the general population [2]. HIV leads to progressive immunodeficiency and increased susceptibility to infections. Unlike many other infectious diseases that only manifest when the CD4 count falls below 200/mm^3^, the risk of TB is already increased during the first year of HIV infection. As a result, TB may be diagnosed long before HIV in co-infected patients [3].

Several studies have found a lower TB treatment success rate among co-infected patients [1, 4–7]. An important intervention that can have a substantial impact on the reduction of mortality and morbidity among co-infected patients is the use of antiretroviral treatment (ART) [1]. In support of this, research has found that the initiation of ART during TB treatment significantly improves the chance of survival [5, 8–15]. For these reasons, integration of TB and HIV services is widely advocated [16–20]. Reciprocal benefits exist for integrating TB and HIV/AIDS services, as adequate TB control contributes to AIDS care, and the prevention of HIV transmission leads to improved TB control. While there is a policy commitment to provide integrated TB and HIV services in South Africa, the reality is that services remain largely fragmented and provided in silos [21].

This study analyses the routinely captured demographic and clinical characteristics of new TB cases (15 years and older), with a treatment outcome recorded between 1 January 2009 and 31 December 2012. It also identifies risk factors for unsuccessful treatment outcomes for those cases co-infected with HIV.

## Methods

### Setting

This study was conducted in the Free State province. In 2012, the TB incidence in the Free State was 708.5 cases per 100,000 population, which was higher than the incidence in the country as a whole – 687.3 cases per 100,000 [21]. HIV prevalence was 19.6% among adults 15-49 years of age, slightly higher than the national prevalence of 17.9% [22]. In 2012, the treatment success rate for co-infected cases in South Africa was 74% [23] and this increased slightly to 76% by 2013 [1], but is still a far cry from the WHO target of 85%.

### Design and population

The study followed a cross-sectional retrospective record review of routinely collected TB data in the Free State electronic TB database (ETR.net) between 2009 and 2012. ETR.net is a Microsoft.net based computer software programme that is based on the recording formats of the World Health Organization (WHO) and International Union Against TB and Lung Disease (IUATLD). It was developed for more efficient and useful collection, compilation and analysis of TB data. ETR.net is the designated software to capture TB data in South Africa and was implemented in 2003 [24]. TB data in the Free State are recorded at primary health care level in a paper-based TB register. These data are then captured on the ETR.net at sub-district level and sent to the Provincial Department of Health where it is aggregated.

The study population was defined as all new TB cases, older than 14 years of age with a treatment outcome recorded between 1 January 2009 and 31 December 2012 in the ETR.Net. A new TB patient is defined as a person who has never had TB treatment or who has taken TB drugs for less than 4 weeks. They may have Gene Xpert, smear or culture positive/negative pulmonary TB (PTB) or extra-pulmonary TB (EPTB) [3]. In keeping with WHO age classifications, adults (i.e. 15 years and older) were included in this analysis. The start date, 2009, was selected as political commitment and increased availability of resources (human and material) resulted in more complete recording of HIV status in the ETR.net from 2009.

### Measures

Duplicate case entries were deleted from the database and patients’ names were removed before the data were extracted for analysis. Cases with “moved/transferred” as a recorded outcome were also excluded from the analysis. Unsuccessful TB treatment outcomes includes cases that had failed treatment, died or defaulted. Treatment “failure” applies to patients whose baseline smear/culture was positive and remains or becomes positive again at five months or later during treatment. This excludes patients who were diagnosed with Rifampicin Resistant-TB or MDR-TB during treatment. “Died” refers to a patient who dies for any reason during the course of TB treatment. “Default” applies to patients whose treatment was interrupted for two consecutive months or more during the treatment period. Successful TB treatment outcomes includes cases who were cured or who had completed treatment. In order to be classified as “cured”, patients had to have a positive baseline smear/culture at the start of treatment and a negative smear/culture negative during the last month of treatment. In addition, they had to have a negative smear/culture at least 30 days prior to completing treatment. Patients who had a positive baseline smear/culture at the beginning of treatment and who completed treatment but did not have a negative smear/culture negative during the last month of treatment or at least thirty days prior to this, are classified as “completed” treatment cases [3].

Information was extracted on socio-demographic variables including sex (male/female), age (15-24/25-34/35-44/45-54/55-64/≥65 years) location by health district (A/B/C/D/E) and year of registration (2009, 2010, 2011, 2012), as well as clinical variables including HIV status (positive/negative/unknown), disease classification (PTB/EPTB/both), pre-treatment sputum smear result (negative/positive/no smear), treatment delay (calculated as the difference in the number of days between notification and treatment initiation: 0-14 days/>14 days), treatment uptake (ART only/CPT only/neither ART or CPT/both ART and CPT), and CD4 count (≤ 200 cells/mm^3^/201-350 cells/mm^3^/≥ 351 cells/mm^3^).

### Analysis

The data were cleaned and analysed using Stata v. 12. Data were described using frequency counts and percentages for categorical variables and means and standard deviations for continuous variables. The outcome variable was unsuccessful TB treatment, which was defined as new cases who had died during treatment, defaulted on or failed treatment. Pearson’s X^2^ test was used to establish any association between independent variables and the outcome variable. Multivariate logistic regression analysis was used to determine which factors were significantly associated with unsuccessful treatment outcomes for TB-HIV co-infected cases. The odds ratios (ORs) together with their corresponding 95% confidence intervals (CIs) were estimated. The significance level considered for this study was 0.05.

### Ethical considerations

Ethical clearance was obtained for the study from the Ethics Committee of the Faculty of Health Sciences, University of the Free State (ECUFS No 1864/2014), and the study was authorised by the Free State Department of Health. The ethics committee granted a waiver for individual patient consent given that this data were aggregated and anonymised at the provincial level.

## Results

**Fig. 1:**
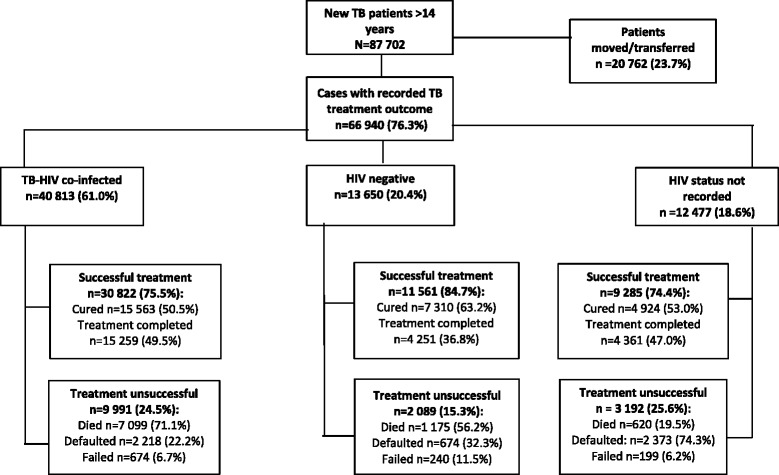
Overview of cases included in the analysis (Free State 1 January 2009- 31 December 2012)

During 2009-2012 there were 87,702 new TB cases among persons 15 years and older in the Free State. Of these, 23.7% were moved/transferred out and as a result excluded from the analysis (see Fig. 1). The proportion of males (50.4%) and females (49.6%) excluded were significantly different from those males (55%) and females (45%) included in the study. The proportions in each age group for cases included and excluded in the analysis were similar except for: 25-34 (29.3% vs 31.3%); 35-44 (28.5% vs 27.7%) and ≥65 (3% vs 3.7%) (95%CI for proportions overlap see Additional file 1). Of the patients with a recorded TB treatment outcome (76.3%): 61% were co-infected with HIV and 18.6% did not have a recorded HIV status. An unsuccessful TB treatment outcome was indicated for 24.5% of the co-infected cases, 15.3% of HIV negative cases and 25.6% of cases that did not have a recorded HIV status. A breakdown of unsuccessful treatment outcomes revealed that for both co-infected cases (71.1%) and HIV negative cases (56.2%), death was the main outcome.

Table 1 illustrates the demographic and clinical profile of all cases stratified by HIV status, as well as those cases that did not have HIV status recorded. In terms of the demographic variables, in the co-infected group there were slightly more males (52.8%) and the average age was 37.4 years (standard deviation ±10.31). There were significant associations amongst the TB-HIV co-infected group in terms of sex, age, district, disease classification being on treatment, CD4 count and an unsuccessful TB treatment outcome (*p* < 0.001). Amongst the HIV negative group, the majority (69.1%) were male and the average age was 39.9 years (standard deviation ±16.56). There were significant associations between sex, age, disease classification, pre-treatment smear result and an unsuccessful TB treatment outcome (*P* < 0.001).

**Table 1:** Demographic and clinical characteristics of all new TB patients who had an unsuccessful TB treatment outcome, stratified by HIV status

Variable	TB-HIV Co-infected *n* = 9991 *n* (%)	95% confidence interval)	HIV negative *n* = 2089 *n* (%)	95% confidence interval	HIV status not recorded *n* = 3192 *n* (%)	95% confidence interval	Total *n* = 15,272 *n* (%)	95% confidence interval
Sex								
Male	5276 (52.8)	51.8-53.8	1444 (69.1)	67.1-71.1	1924 (60.3)	58.6-62.0	8644 (56.6)	55.8-57.4
Female	4715 (47.2)	46.2-48.2	645 (30.9)	28.9-32.6	1268 (39.7)	38.2-41.4	6628 (43.4)	42.6-44.2
Age group								
15-24	816 (8.2)	7.6-8.7	318 (15.2)	13.7-16.8	268 (8.4)	7.4-9.4	1402 (9.2)	8.7-9.6
25-34	3215 (32.2)	31.3-33.1	342 (16.4)	14.8-17.9	837 (26.2)	24.7-27.7	4394 (28.8)	28.1-29.5
35-44	3287 (32.9)	32.0-33.8	322 (15.4)	13.9-167.0	805 (25.2)	23.7-26.7	4414 (28.9)	28.2-29.6
45-54	1903 (19.0)	18.3-19.8	395 (18.9)	17.2-20.6	685 (21.5)	20.0-22.9	2983 (19.5)	18.9-20.2
55-64	643 (6.4)	5.9-6.9	389 (18.6)	16.9-20.3	362 (11.3)	10.2-21.4	1394 (9.1)	8.7-9.6
≥65	127 (1.3)		323 (15.5)	13.9-17.0	235 (7.4)	6.5-8.3	685 (4.5)	4.1-4.8
District								
A	1786 (17.9)	17.1-18.6	3 66 (17.5)	15.9-19.2	448 (14.0)	12.8-15.2	2600 (17.0)	16.4-17.6
B	2449 (24.5)	23.7-25.4	471 (22.5)	20.8-24.3	482 (15.1)	13.9-16.3	3402 (22.3)	21.6-22.9
C	2649 (26.5)	25.6-27.4	517 (24.8)	22.8.9-26.6	1007 (31.6)	29.9-33.1	4173 (27.3)	26.728.0
D	2620 (26.2)	25.4-27.1	540 (25.9)	234.0-27.7	1104 (34.6)	32.9-36.2	4264 (27.9)	27.2-28.6
E	487 (4.9)	4.4-5.3	195 (9.3)	8.1-10.6	151 (4.7)	4.0-5.5	833 (5.5)	5.1-5.8
Disease classification								
Both	187 (1.9)	1.6-2.1	26 (1.2)	0.7-1.7	42 (1.3)	0.9-1.7	255 (1.7)	1.5-1.9
Extra-pulmonary	2135 (21.4)	20.6-22.2	307 (14.7)	13.2-16.2	832 (26.1)	24.5-27.6	3274 (21.4)	20.8-22.1
Pulmonary	7669 (76.7)	75.9-77.6	1756 (84.1)	82.5-85.6	2318 (72.6)	71.0-74.2	11,743 (76.9)	76.2-77.6
Pre-treatment sputum smear result^a^								
Sputum smear positive	4346 (61.2)	60.0-62.3	1083 (65.5)	63.2-67.8	1196 (67.4)	65.2-69.6	6625 (62.9)	62.0-63.8
Sputum smear negative	2760 (38.8)	37.7-40.0	571 (34.5)	32.2-36.8	578 (32.6)	30.3-34.8	3909 (37.1)	36.2-38.0
Treatment delay^b^								
0-14 days	4772 (83.0)	82.1-84.1	1166 (85.3)	83.4-87.2	1251 (85.5)	83.7-87.3	7189 (83.8)	83.1-84.6
15+ days	974 (17.0)	16.0-17.9	201 (14.7)	12.8-16.5	212 (14.5)	12.7-16.3	1387 (16.2)	15.4-17.0
On ART/CPT								
Neither on ART nor CPT only	1292 (12.9)	12.3-13.6	NA	NA	NA	NA	1292 (12.9)	12.3-13.6
On ART only	367 (3.7)	3.3-4.0	NA	NA	NA	NA	367 (3.7)	3.3-4.0
On CPT only	4202 (42.1)	41.1-43.0	NA	NA	NA	NA	4202 (42.1)	41.1-43.0
On both ART and CPT	4130 (41.3)	40.4-42.3	NA	NA	NA	NA	4130 (41.3)	40.4-42.3
CD4 count								
1-200	4211 (75.9)	74.7-77.0	NA	NA	NA	NA	4211 (75.9)	74.7-77.0
201-350	839 (15.1)	14.2-16.1	NA	NA	NA	NA	839 (15.1)	14.2-16.1
351+	502 (9.0)	8.2-9.8	NA	NA	NA	NA	502 (9.0)	8.2-9.8

*NA* not applicable.

^a^HIV-positive *n* = 7106, HIV-negative *n* = 1654, HIV status unknown *n* = 1774

^b^HIV-positive *n* = 5746, HIV-negative *n* = 1367, HIV status unknown *n* = 1463

With regard to the clinical profile of the co-infected cases, seven in ten cases (76.7%) had pulmonary TB (PTB), 61.2% had a positive pre-treatment smear, 42.1% were on CPT and 41.3% were on both ART and CPT. Furthermore, the majority of cases (75.9%) had a low CD4 count of <201 cells/mm^3^.There were significant associations between disease classification, being on treatment, CD4 count, and an unsuccessful treatment result (*P* < 0.001). The clinical profile of the HIV negative cases revealed that four in five cases (84.1%) had PTB and two-thirds (67.4%) had a positive pre-treatment smear result. There were significant associations between disease classification, pre-treatment smear results and an unsuccessful TB treatment outcome (*p* < 0.001).

**Fig. 2:**
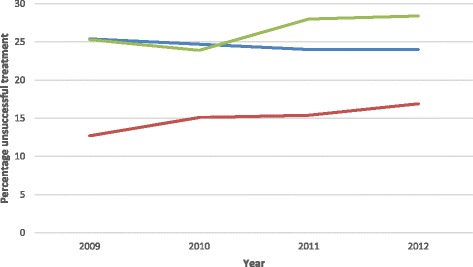
Percentage of unsuccessful TB treatment outcomes by HIV status.  Co-infected,  HIV negative,  HIV status not recorded

**Fig. 3:**
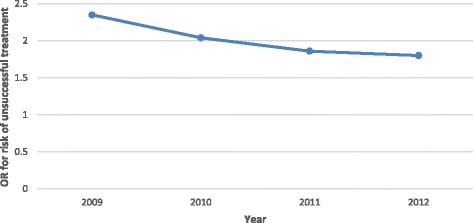
Risk of unsuccessful treatment for HIV positive cases by year of diagnosis (adjusted for age and district)

Despite the slight decrease in unsuccessful TB treatment outcomes for the co-infected cases (25.4% in 2009 and 24% in 2012) and a slight increase in unsuccessful treatment outcomes among HIV negative cases (12.7% in 2009 and 16.9% in 2012), unsuccessful treatment outcomes remained constantly higher among the co-infected group (see Fig. 2). Univariate odds ratios revealed that in 2009, the TB-HIV co-infected cases were 2.35 times more at risk for an unsuccessful TB treatment outcome (OR: 2.35; CI: 2.06-2.69) than the HIV negative cases; however, this figure decreased to 1.8 times in 2012 (OR: 1.80; CI: 1.63-1.99) (see Fig. 3).

Univariate odds ratios (Table 2) further identified the following risk factors for an unsuccessful TB treatment outcome: persons 65 years and older were 1.81 times more likely to have an unsuccessful TB treatment outcome (OR: 1.81; CI: 1.33-2.47) than 15-24 year olds; patients in the District D were 1.17 times (highest) more likely to have an unsuccessful TB treatment outcome (OR:1.17; CI: 1.07-1.23) than those in District A (lowest); and patients taking CPT only were 1.22 times more likely to have an unsuccessful outcome (OR: 1.22; CI: 1.00-1.49) than those taking ART only. Furthermore, females were less likely to have an unsuccessful TB treatment outcome (OR: 0.89; CI: 0.84-0.95) compared to their male counterparts. Persons with a CD4 count of ≥351 cells/mm^3^ (OR: 0.46; CI: 0.42-0.52) were also less likely to have an unsuccessful TB treatment outcome compared to those with a CD4 count ≤200 cells/mm^3^.

**Table 2:** Factors associated with unsuccessful TB treatment outcomes for TB-HIV co-infected cases

	Univariate Odds Ratio (95% CI)	Adjusted Odds Ratio (95% CI)
Sex		
Male (ref)	1	1
Female	0.89 (0.84-0.95)	0.93 (0.88-0.99)
Age		
15-24 years (ref)	1	1
25-34 years	0.95 (0.85-1.07)	0.86 (0.77-0.97)
35-44 years	0.99 (0.89-1.12)	0.89 (0.79-1.00)
45-54 years	1.08 (0.95-1.22)	0.99 (0.87-1.12)
55-64 years	1.28 (1.08-1.50)	1.22 (1.04-1.45)
65+ years	1.81 (1.33-2.47)	1.71 (1.25-2.35)
District		
A	1	1
B	0.95 (0.86-1.04)	0.94 (0.85-1.03)
C	0.90 (0.82-0.99)	0.82 (0.75-0.91)
D	1.17 (1.07-1.23)	1.15 (1.05-1.28)
E	0.78 (0.68-0.90)	0.88 (0.77-1.02)
Year		
2009 (ref)	1	
2010	1.00 (0.90-1.10)	
2011	0.98 (0.89-1.07)	
2012	0.97 (0.88-1.06)	
On ART/CPT		
ART only	1	1
CPT only	1.22 (1.00-1.49)	1.28 (1.05-1.57)
Neither ART nor CPT	0.87 (0.68-1.10)	0.97 (0.76-1.24)
Both ART and CPT	0.80 (0.66-0.97)	0.75 (0.61-0.91)
CD4 results		
1-200	1	1
201-350	0.56 (0.52-0.61)	0.55 (0.51-0.60)
351+	0.46 (0.42-0.52)	0.40 (0.36-0.44)

**Fig. 4:**
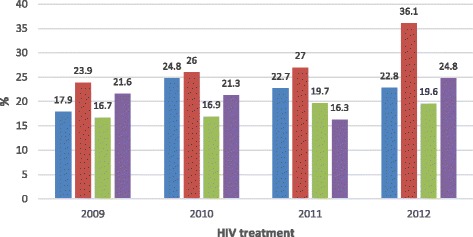
HIV treatment status of cases with unsuccessful TB treatment outcomes.  ART,  CPT,  ART&CPT,  No treatment

After adjusting for other variables (sex, age, location/district, on treatment and CD4 count) (Table 2), the factors that were significantly associated with unsuccessful TB treatment outcomes were: being 55 years and older; receiving treatment in District D, and taking CPT only. Persons aged 55-64 years were 1.22 times more likely to have an unsuccessful treatment outcome (AOR: 1.22; CI: 1.04-1.45), and persons 65 years and older were 1.71 times more likely to have an unsuccessful treatment (AOR: 1.71; CI: 1.25-2.35) outcome than patients in the 15-24 year age group. Patients receiving treatment in District D were 1.15 times more likely to have an unsuccessful treatment outcome (AOR: 1.15; CI 1.05-1.28) than patients receiving treatment in District A. Those cases taking CPT, were 1.28 times more likely to have an unsuccessful treatment outcome (AOR: 1.28; CI: 1.05-1.57) than cases taking ART. Over the four year period, the percentage of cases with unsuccessful TB treatment outcomes who were only on CPT Increased from 23.9% in 2009 to 36.1% in 2012, with those cases on both ART and CPT constantly having a lower rate of unsuccessful TB treatment outcomes (see Fig. 4).

Females were less likely to have an unsuccessful treatment outcome (AOR: 0.93; CI: 0.88-0.99), indicating that males were more at risk for an unsuccessful treatment outcome. Persons with a CD4 count 201-350 cells/mm^3^ (AOR: 0.55; CI: 0.51-0.60) as well as those with a CD4 count of ≥351 cells/mm^3^ (AOR: 0.40; CI: 0.36-0.44) were also less likely to have an unsuccessful treatment outcome than cases with a low CD4 count (i.e. ≤ 200 cells/mm^3^). This implies that a low CD4 count increases the risk for an unsuccessful treatment outcome.

## Discussion

Of the 87,702 new adult TB cases in the Free State (2009-2012), 22.8% had an unsuccessful TB treatment outcome, the majority of these cases (18.3%) were co-infected with HIV. As HIV is one of the major factors fuelling the growing TB epidemic [20, 25, 26] - 61% of the TB cases in the province were co-infected with HIV/AIDS, which was slightly lower than the 65% average reported over the same period for South Africa [27] - the analysis of data in this paper was stratified by HIV status. During the study period (2009-2012), the HIV status of the majority (81.4%) of cases was known. In line with the WHO [19] recommendation that all patients with (presumptive) TB be offered HIV testing, it is encouraging to note that by 2014, there was a substantial improvement in the country as a whole, with 93% of TB patients knowing their HIV status [1]. Similarly to other studies [1, 4–7] we found significant differences in treatment outcomes between patients who were HIV-negative and HIV-positive. Over the four year study period, 24.5% of HIV-negative patients compared to 15.3% of HIV-positive patients had an unsuccessful treatment outcome.

The poor treatment success rate could partly be explained by the fact that only by 2011 were 60% and more co-infected cases on ART. This is in line with national guidelines, which changed over time, regarding the eligibility criteria for TB-HIV co-infected patients to be on ART. Only by April 2013 were all co-infected patients eligible for ART irrespective of CD4 count [28] despite numerous research studies that found that survival/treatment success among co-infected patients was strongly associated with patients being on ART [5, 8–15]. Furthermore, it has been reported that early ART initiation (i.e. two weeks after starting TB treatment) in TB patients significantly improves survival among co-infected patients [10].

National guidelines [3] and research findings [13, 14, 29] also indicate that CPT decreases morbidity and mortality in TB/HIV co-infected patients. Despite WHO [30] guidelines that CPT should be initiated prior to ART, our study found that patients who were only on CPT and not on a combination of ART and CPT, were at an increased risk of an unsuccessful treatment outcome. A possible explanation for this is that three quarters of the cases had a CD4 count <201, and as a result would have been more severely ill, requiring ART. Furthermore, patients receiving ART have had drug readiness training to ensure adherence to treatment, which may encourage them to be more compliant to strict treatment regimens than patients who have not undergone such training. Therefore, co-infected cases should be started on ART immediately, as CPT alone is not a strong factor for securing a successful TB treatment outcome. This is in line with recent international and national guidelines regarding the treatment of HIV in co-infected patients.

In addition to ART and CPT, we found that patients with a higher CD4 count were less likely to have an unsuccessful TB treatment outcome c.f. [13, 15]. As with a number of other research studies [5, 31–33], we also found that females were less likely to have an unsuccessful TB treatment outcome, although Ejeta et al. [14] did not find a significant association between treatment success and sex. Mukherejee et al. [33], who conducted a retrospective record-based study between 1999 and 2005 in India, postulated that better treatment outcomes in females may reflect biological differences in addition to health system-related factors. A qualitative study by Carlsson et al. [34] in Burundi reported that nurses found women more likely to comply with TB treatment than men as they were more emotional than men, cared for their families and were afraid of infecting others.

To our knowledge, this is the first large scale study to investigate unsuccessful TB treatment outcomes in the Free State Province. The study is not only beneficial to the Free State but also to other similar settings grappling with improving TB treatment outcomes, particularly for co-infected patients. In this regard, TB surveillance, through ongoing and systematic collection, analysis, interpretation and dissemination of information, plays a critical role in informed decision making. As such, surveillance is one of the five core components in the original 1994 WHO *Framework for Effective Tuberculosis Control* (the WHO DOTS strategy) [35]. More specifically, the assessment of TB treatment outcomes and subsequent analysis of factors associated with unsuccessful treatment outcomes is of vital importance in monitoring the success of the TB treatment programme and identifying high risk and vulnerable populations, especially in a country such as South Africa where both the TB and HIV epidemics are rampant.

## Limitations

The main limitation of the study, as with other retrospective record reviews [7, 14, 31], was that the data were analysed retrospectively; and in essence this meant that it was not possible to correct any obvious errors in the data, nor could missing data be captured. As an existing set of routinely collected data from the ETR.net were used, this excluded other important variables that could have had an influence on TB treatment success of co-infected patients. For example, socio-cultural (e.g. stigma, cultural beliefs, education) economic (e.g. employment, occupation, income) and individual patient (e.g. alcohol use/abuse and smoking) factors. Furthermore, key information such as the dates ART and/or CPT commenced were not recorded nor was information on MDR TB. As a result, this study could not measure the effect of MDR TB as a risk factor for unsuccessful TB treatment. MDR TB is not recorded in the ETR.net, but in a separate MDR TB electronic case register.

## Conclusions

Unsuccessful TB treatment outcomes remained problematic in the Free State over the study period, especially among the TB-HIV co-infected patients. Despite all co-infected patients being eligible for ART, approximately 20% of those co-infected were not on such treatment. The importance of TB-HIV/AIDS treatment integration becomes evident as patients on a combination of ART and CPT, and with a higher CD4 count are more likely to have a successful TB outcome. The TB control programme in the Free State is clearly faced with the challenge of rolling out ART to all TB patients. In light of the 90:90:90 HIV treatment strategy adopted by South Africa (i.e. by 2020, 90% of all people living with HIV will know their HIV status; 90% of all people with diagnosed HIV infection will receive sustained ART; and 90% of all people receiving antiretroviral therapy will have viral suppression) [36], this study identified risk groups requiring urgent programmatic attention, including older patients and males.

## Additional file

Additional file 1: Demographic characteristics of included and excluded cases in the analysis. The proportion of males (50.4%) and females (49.6%) excluded were significantly different from those males (55%) and females (45%) included in the study. The proportions in each age group for cases included and excluded in the analysis were similar except for: 25-34 (29.3% vs 31.3%); 35-44 (28.5% vs 27.7%) and ≥65 (3% vs 3.7%) (95% CI for proportions overlap see Additional file 1). (DOCX 13 kb)

## Abbreviations

ART: Antiretroviral treatment
CIs: Confidence intervals
CPT: Cotrimoxazole prophylaxis
EPTB: Extra-pulmonary
ETR.net: Electronic TB database/register
HIV: Human immune deficiency virus
MDG: Millenium development goal
MDR-TB: Multidrug resistant tuberculosis
NDoH: National Department of Health
ORs: Odds ratios
PTB: Pulmonary TB
TB: Tuberculosis
UN: United Nations
UNAIDS: Joint United Nations Programme on HIV/AIDS
WHO: World Health Organization
