# Supplementary material for: Unsuccessful TB treatment outcomes with a focus on HIV co-infected cases: a cross-sectional retrospective record review in a high-burdened province of South Africa
Source: BMC Health Serv Res. 2017 Jul 10;17:470. doi: 10.1186/s12913-017-2406-x (PMC5504727; doi:10.1186/s12913-017-2406-x)
Supplement: Additional file 1: — Demographic characteristics of included and excluded cases in the analysis. The proportion of males (50.4%) and females (49.6%) excluded were significantly different from those males (55%) and females (45%) included in the study. The proportions in each age group for cases included and excluded in the analysis were similar except for: 25-34 (29.3% vs 31.3%); 35-44 (28.5% vs 27.7%) and ≥65 (3% vs 3.7%) (95% CI for proportions overlap see Additional file 1). (DOCX 13 kb) [file 12913_2017_2406_MOESM1_ESM.docx]

**Demographic characteristics of included and excluded cases in the analysis**

| **Demographics** | **Included cases**  **N=66 940** | | **Excluded cases**  **N=20 762** | |
| --- | --- | --- | --- | --- |
|  | **n(*%)*** | ***CI*** | **n*(%)*** | ***CI*** |
| **Sex** | | | | |
| Male | 36 811 (55.0) | 54.61-55.40 | 10 459 (50.4) | 49.70-51.06 |
| Female | 30 129 (45.0) | 44.63-45.39 | 10 303 (49.6) | 48.94-50.30 |
| **Age** | | | | |
| 15-24 | 8 515 (12.7) | 12.47-12.97 | 2 646 (12.74) | 12.29-13.20 |
| 25-34 | 19 588 (29.3) | 28.91-29.61 | 6 503 (31.3) | 30.70-31.95 |
| 35-44 | 19 099 (28.5) | 28.19-28.87 | 5746 (27.7) | 27.07-28.28 |
| 45-54 | 12 677 (18.9) | 18.64-19.23 | 3 498 (16.8) | 16.34-17.36 |
| 55-64 | 5 079 (7.6) | 7.37-7.79 | 1 599 (7.7) | 7.33-8.06 |
| ≥65 | 1 982(3.0) | 2.83-3.10 | 770 (3.7) | 3.45-3.70 |
